# Effectiveness of Occupational Therapy Interventions on Activities of Daily Living, Cognitive Function, and Physical Function in Middle-Aged and Older People with Chronic Stroke: A Systematic Review with Meta-Analysis

**DOI:** 10.3390/jcm14072197

**Published:** 2025-03-24

**Authors:** Edgar Vásquez-Carrasco, Pía Jamett-Oliva, Jordan Hernandez-Martinez, Cristóbal Riquelme-Hernández, Francisca Villagrán-Silva, Braulio Henrique Magnani Branco, Cristian Sandoval, Pablo Valdés-Badilla

**Affiliations:** 1 School of Occupational Therapy, Faculty of Psychology, Universidad de Talca, Talca 3465548, Chile; edgar.vasquez@utalca.cl (E.V.-C.); pjamett21@alumnos.utalca.cl (P.J.-O.); 2 Centro de Investigación en Ciencias Cognitivas, Faculty of Psychology, Universidad de Talca, Talca 3465548, Chile; 3 Department of Physical Activity Sciences, Universidad de Los Lagos, Osorno 5290000, Chile; jordan.hernandez@ulagos.cl; 4 G-IDyAF Research Group, Department of Physical Activity Sciences, Universidad de Los Lagos, Osorno 5290000, Chile; 5 Programa de Investigación en Deporte, Sociedad y Buen Vivir, Universidad de los Lagos, Osorno 5290000, Chile; 6 Departamento de Salud, Universidad Arturo Prat, Victoria 4720000, Chile; crriquelme@unap.cl; 7 Programa de Doctorado en Ciencias Morfológicas, Facultad de Medicina, Universidad de La Frontera, Temuco 4811230, Chile; f.villagran04@ufromail.cl; 8 Postgraduate Program in Health Promotion, Cesumar University, Maringá 87050-390, Paraná, Brazil; braulio.branco@unicesumar.edu.br; 9 Escuela de Tecnología Médica, Facultad de Salud, Universidad Santo Tomás, Los Carreras 753, Osorno 5310431, Chile; 10 Departamento de Medicina Interna, Facultad de Medicina, Universidad de La Frontera, Temuco 4811230, Chile; 11 Núcleo Científico y Tecnológico en Biorecursos (BIOREN), Universidad de La Frontera, Temuco 4811230, Chile; 12 Department of Physical Activity Sciences, Faculty of Education Sciences, Universidad Católica del Maule, Talca 3530000, Chile; 13 Sports Coach Career, School of Education, Universidad Viña del Mar, Viña del Mar 2520000, Chile

**Keywords:** activities of daily living, aged, occupations, occupational therapy, stroke

## Abstract

**Background:** Occupational therapy (OT) interventions on activities of daily living (ADL), cognitive functions, and physical function in middle-aged and older people with chronic stroke. **Methods**: A systematic review search until November 2024 using five generic databases: PubMed/Medline, Web of Science, Scopus, ScienceDirect, and OT seeker. The PRISMA checklist, RoB 2 (Cochrane, London, UK), and GRADEpro (Evidence Prime Inc., Hamilton, CA) tools assessed the evidence’s methodological quality and certainty. The protocol was registered in PROSPERO (code: CRD42024568225). **Results:** Of 1733 records were identified across the databases, nine studies were analyzed using the PICOS format. The meta-analysis revealed significant improvements in independent performance of activities of daily living (ADL), as measured by the Canadian Occupational Performance Measure (COPM), in favor of the experimental groups (*p* = 0.03). No significant differences were found for the other variables analyzed. **Conclusions**: Performance on ADLs improved significantly according to the COPM, whereas no significant improvements in cognitive or physical function were observed among middle-aged and older chronic stroke survivors. Individual studies highlight the potential benefits of OT interventions that combine cognitive, motor, and technological approaches, such as virtual reality and brain stimulation.

## 1. Introduction

Stroke represents a significant public health challenge worldwide and ranks among the most disabling diseases [1]. It affects 15 million people annually, with 70% of cases and 87% of related deaths occurring in low- and middle-income countries [2]. Stroke results in neurological and neuropsychological sequelae that impair cognitive, sensory, and motor functions [3].

Between 35% and 40% of stroke survivors experience limitations in basic activities of daily living (ADL) six months after the event, and approximately 40% live with moderate to severe disabilities [4]. One of the professions that deals with the rehabilitation of post-stroke individuals is (OT), a client-centered discipline focused on promoting health and well-being through occupation with the primary goal of facilitating participation in daily activities by enhancing individual abilities or adapting occupations and environments to support engagement [5]. Globally, Occupational therapy (OT) is crucial in chronic stroke rehabilitation, significantly enhancing ADLs, health-related quality of life, and restoring survivors’ independence and functionality [6]. Active OT significantly reduced the length of hospitalization (*p* < 0.001) and improved functional independence (*p* < 0.001) and neurological status (*p* < 0.001) [7]. Interventions with strong strength of evidence for improving performance in activities of daily living and functional mobility include mirror therapy, task-oriented training, mental imagery, balance training, and self-management strategies [8]. A meta-analysis concluded that activity-oriented OT significantly improves ADL performance (*p* < 0.001) and physical function (*p* < 0.001) in stroke patients [7]. Tedla et al. [9] used a meta-analysis to evaluate the efficacy of constraint-induced movement therapy in patients who have suffered a stroke; the results showed significant improvements in balance (*p* = 0.01) compared to other therapies. Cognitive function is an essential part of treatment; a meta-analysis examined the effects of OT on cognitive function after stroke, and the main findings indicated a clinically significant improvement in global cognitive performance (*p* = 0.0004) [10].

New directions in OT for stroke rehabilitation could include the integration of technologies such as virtual reality (VR) training to enhance cognitive and functional recovery, which is presented as a beneficial tool when used in conventional treatments to improve motor and cognitive functions of people who have suffered a stroke and encourages adherence to the interventional rehabilitation process through OT [11]. These technologies can enable the development of personalized programs using virtual simulations of daily activities, promoting patient independence and social reintegration [12]. Neuroscientific methods, such as non-invasive brain stimulation, could also complement OT treatments by enhancing brain plasticity and functional recovery [13]. The incorporation of such technologies facilitates intensive, personalized rehabilitation by providing precise motor training and enabling continuous monitoring of patient progress [14]. Therefore, this systematic review with meta-analysis aimed to evaluate and synthesize the scientific evidence of OT interventions on ADL, cognitive functions, and physical function in middle-aged and older people with chronic stroke.

## 2. Methods

### 2.1. Protocol and Registration

This systematic review with meta-analysis followed the Cochrane Collaboration methodology [15] and adhered to the PRISMA checklist and flowchart standards [16]. The review was registered in the PROSPERO database under registration number CRD42024568225.

### 2.2. Eligibility Criteria

The inclusion criteria were original, randomized controlled trials (RCTs) with no language or publication date constraints were eligible until November 2024. Conference abstracts, books, chapters, editorials, letters to the editor, procedures, reviews, case reports, and opinions were excluded. Table 1 shows how the PICOS framework (Population, Intervention, Comparator, Outcome, and Study Design) was used to analyze the studies.

### 2.3. Information and Database Search Process

Five generic databases were searched: PubMed/Medline, Web of Science (core collection), Scopus, ScienceDirect, and OTseeker. The search included MeSH terms and free-text keywords. For example, the search used the following terms: (“Activities of Daily Living” OR “Leisure Activities” OR “Social Participation” OR “Sexual Behavior” OR “Community Participation”) AND (“Stroke” OR “Cerebrovascular Disorders” OR “Brain Vascular Disease” OR “Cerebrovascular Accident” OR “Brain Vascular Accident” OR “Cerebrovascular Stroke”) AND (“adults” OR “middle-aged” OR “middle-aged” OR “elderly” OR “older adults” OR “older people” OR “older subject” OR “aging” OR “ageing” OR “aged”).

Included articles and the inclusion/exclusion criteria were reviewed by two independent experts with the following qualifications: (i) a doctorate in health-oriented sciences and (ii) peer-reviewed publications in journals with an impact factor (Journal Citation Reports^®^). Experts were not provided with the search strategy to minimize bias. A final database search on 30 November 2024 aimed to identify relevant errata or retractions related to the included studies.

### 2.4. Study Selection and Data Collection Process

Studies were imported into Mendeley Reference Manager (Version 2.116.1). Two reviewers (E.V.-C. and P.J.O.) independently screened titles, abstracts, and full texts, removing duplicates. Articles meeting the inclusion criteria were reassessed, and reasons for excluding others were documented. A third reviewer (P.V.-B.) verified the data selection and extraction process.

### 2.5. Methodological Quality Assessment

The methodological quality and level of evidence were assessed using the Oxford Centre for Evidence-Based Medicine scale [17]. Only level 1a studies, defined as randomized controlled trials (RCTs), were included. Studies at levels 1b through 5 were excluded. RCTs were downgraded if issues related to bias, consistency, accuracy, precision, or transparency of results were identified [17].

### 2.6. Data Extraction

Data from the selected studies were collected using a standardized template in Microsoft Excel^®^ (version 16.81), adhering to Cochrane guidelines [15]. Two researchers, E.V.-C. and P.J.O., independently extracted the data and subsequently compared their findings to ensure accuracy. A third reviewer (P.V.-B.) oversaw the process to maintain quality control. The extracted variables comprised authors, country of origin, study design, sample size, group sizes (*n*), mean age (in years), types of intervention and control, training volume (including frequency, duration, and intensity), and assessments of activities of daily living (ADL), cognitive function, physical function, and the primary outcome.

### 2.7. Risk of Bias Assessment

Use the ROB™ tool (Cochrane, London, UK) to assess the risk of bias of RCTs [18]. Two reviewers (E.V.-C. and P.J.-O.) initiated the analysis, and the analysis was subsequently reviewed by a third author (P.V.-B.). Any disagreements were resolved through further review and approval.

### 2.8. Meta-Analysis Measures

For meta-analysis, standardized mean difference (SMD) and mean difference (MD) were used as effect sizes calculated using Comprehensive Meta-Analysis Software (RevMan 5.4). Set the significance level at *p* < 0.05. Random effects models, specifically the Der Simonian-Laird method, are used to pool data from studies with similar findings, considering differences in the true results of the study. Heterogeneity was assessed using the Cochrane Q test and the I² statistic, and the following levels were used for heterogeneity: <25% (low), 25–50% (moderate), and >50% (high) [19]. Egger’s regression test was used to identify potential reporting bias [20].

### 2.9. Certainty of Evidence

The GRADE (Evidence Prime Inc., Hamilton, ON, Canada) framework [18] was used to evaluate the certainty of evidence. Evidence started as high, given the inclusion of RCTs, but could be downgraded due to concerns about bias, consistency, precision, or publication bias. Two reviewers (E.V.-C. and P.J.-O.) conducted independent assessments, with disagreements resolved by consensus involving a third reviewer (P.V.-B.).

## 3. Results

**Figure 1:**
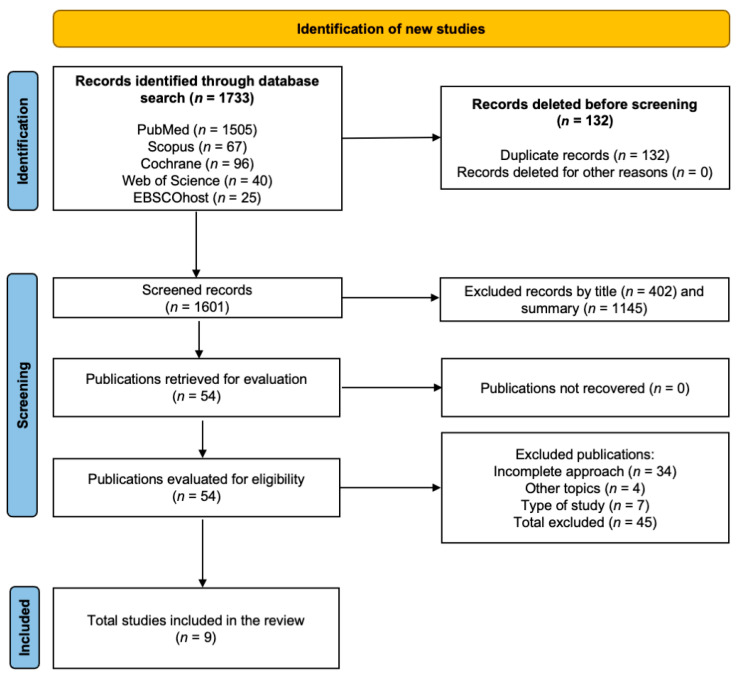
PRISMA flowchart.

A total of 1733 studies were identified through the database search, of which 132 were excluded due to duplication. Of the remaining 1601 records, 1547 were excluded after screening titles and abstracts for relevance. Following a full-text review of 54 references, 45 studies were excluded for not meeting the predetermined inclusion criteria: thirty-four for including incomplete approaches, four for addressing unrelated topics, and seven for not being RCTs. Ultimately, nine studies [21,22,23,24,25,26,27,28,29] were included in the analysis, focusing on interventions for chronic stroke survivors. The search results are illustrated in a flowchart adhering to PRISMA guidelines (Figure 1).

### 3.1. Methodological Quality

The quality of the research evidence included in this meta-analysis systematic review was high. All nine studies were RCTs [21,22,23,24,25,26,27,28,29] and represent the highest level of evidence (level 1a) on the Oxford scale. The study design minimizes the risk of bias and provides a solid basis for assessing the effectiveness of the intervention.

### 3.2. Risks of Bias Within Studies

**Figure 2:**
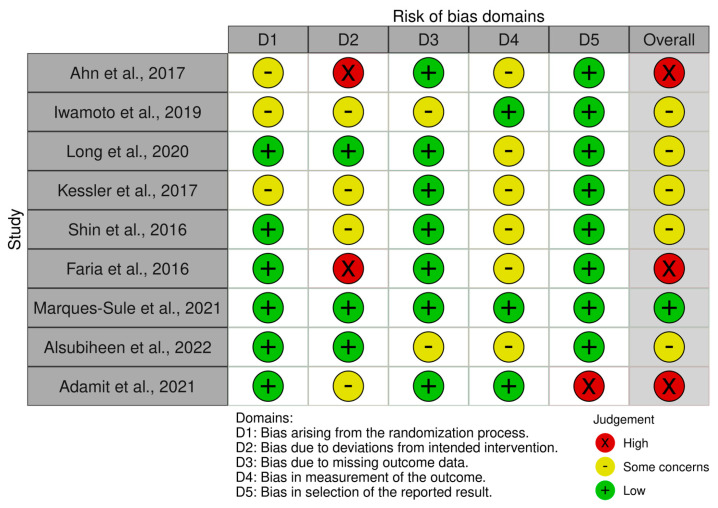
Risk of bias tools: traffic lights chart [21,22,23,24,25,26,27,28,29].

**Figure 3:**
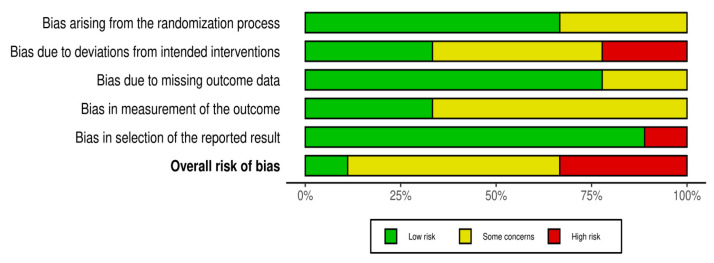
Risk of bias tools: Summary chart by domain.

One study was assessed as having a low risk of bias [27], while five studies were found to have some risk of bias concerns [22,23,24,25,28]. Three studies were classified as having a high risk of bias [21,26,29]. Overall, this suggests that all studies were at risk of bias, as most studies found concerns, and our study found these concerns. Take the risk. Figure 2 and Figure 3 illustrate the risk of bias assessment.

### 3.3. Characteristics of the Studies

Among the nine studies reviewed, interventions varied: VR training, robotic-assisted rehabilitation, cognitively targeted strategies, and hybrid programs combining physical and cognitive training.

Various studies highlight the effectiveness of different interventions in enhancing ADL performance in stroke rehabilitation and related contexts. The Canadian Occupational Performance Measure (COPM) improved ADL satisfaction and performance [21,29]. Assistive technologies like the Hybrid Assistive Limb and RAPAEL Smart Glove™ (Neofect Co. Ltd., Seongnam, Gyeonggi-do, South Korea) enhanced motor functions and movement quality [22,25]. VR training systems, including Doctor Kinetic (Rehab-Robotics, Amsterdam, Netherlands), Nintendo Wii (Nintendo, Kyoto, Japan), and ADL simulations, demonstrated improved balance, mobility, cognitive functions, and strength [23,26,27]. Additionally, specialized programs like OPC-Stroke and Functional and Cognitive Occupational Therapy (FaCoT) led to notable gains in ADL performance and user satisfaction [24,29]. These findings underline the potential of tailored rehabilitation technologies and strategies to optimize ADL outcomes. Detailed descriptions of each study and their outcome measures are provided in Table 2.

### 3.4. Sample Characteristics

The total population included in this systematic review with meta-analysis consisted of 337 middle-aged and older people chronic stroke survivors, of whom 50.4% were female, with a mean age of 57.6 years. Sample sizes ranged from 18 participants [26] to 66 participants [29], reflecting the diversity of intervention strategies.

### 3.5. Dosages and Interventions Performed

The studies included a variety of cognitive and physical interventions aimed at improving ADLs, cognitive function, and physical function among chronic stroke survivors. Cognitive training programs, such as Hybrid Assistive Limb combined with OT and VR training, primarily target cognitive performance and physical function [22,26]. Individualized exercise programs, such as the Cognitive Orientation to Daily Occupational Performance approach and Smart Glove therapy, as well as multicomponent interventions combining physical activity with cognitive training, were shown to improve both ADLs and cognitive function performance significantly [21,25,28].

The interventions varied in duration and frequency, ranging from 4 weeks with three to five 30-minute sessions per week to protocols lasting up to 16 weeks with multiple sessions, each lasting 40 to 60 min [21,22,23,29]. Most programs were delivered at moderate intensity.

### 3.6. Activities of Daily Living

**Figure 4:**
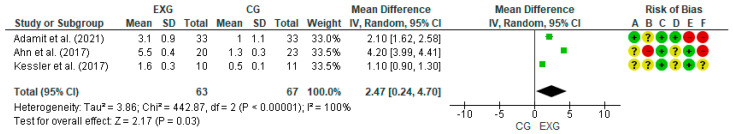
The experimental group has an effect compared to the control group on the following outcomes: MT. Squares indicate the study-specific effect estimate. Bars indicate the width of the corresponding 95% confidence interval. The diamond is centered on the summary effect estimate, and the width indicates the corresponding 95% confidence interval. EXG: experimental group; CG: control group; +: low; ?: some concerns; -: high [21,24,29].

Several studies employed the COPM to measure improvements in ADLs. A meta-analysis revealed a significant improvement in motor training was reported for the experimental groups (EG) concerning control groups (CG; SMD = 2.47; 95% CI = 0.24 to 4.70; I² = 100%; *p* = 0.03). These results are presented in Figure 4.

Individual study results highlight significant improvements in ADLs after various interventions. For example, Ahn et al. [21] reported improved ADL performance and satisfaction, as measured by the COPM-P and COPM-S (*p* < 0.001), using the Cognitive Orientation to Daily Occupational Performance approach. Iwamoto et al. [22] demonstrated improvements in upper body dressing (Functional Independence Measure; *p* = 0.046) and motor activity (*p* < 0.05) in the Hybrid Assistive Limb group. Similarly, Long et al. [23] found an increase in ADL independence with VR training, as indicated by the modified Basel performance index (*p* < 0.000). Marquis de Soule et al. [27] noted a significant improvement in the Barthel index (*p* < 0.01) and Frenchay index (*p* < 0.001) after a VR training intervention. These findings suggest the potential of a new treatment to improve ADL outcomes in stroke survivors.

### 3.7. Cognitive Function

Planned meta-analyses were not possible due to heterogeneity in assessment; therefore, no change favoring EG over CG. However, the reviewed studies showed mixed results on cognitive function. Iwamoto et al. [22] reported no significant changes in the Mini-Mental State assessment after combining Hybrid Assistive Limb and OT. Long et al. [23] reported improvements in the Mini-Mental State (*p* < 0.050) after the use of VR training in combination with rehabilitation. Faria et al. [26] also found improvements in global cognitive function with a significant increase in attention (*p* = 0.040) and memory (*p* = 0.050) in the VR training intervention group. In the study by Kessler et al. [24], no significant improvements in the Montreal Cognitive Assessment were reported (*p* = 0.070). Kessler et al. [24] evaluated OPC-Stroke, a program combining usual care with vocational and professional training. The intervention lasted 4 to 16 weeks, with three to five sessions per week lasting 20 to 60 min each.

### 3.8. Physical Function

A planned meta-analysis could not be conducted due to the heterogeneity assessment; therefore, there is no change favoring EG over CG. The reviewed studies have shown the positive effects of OT interventions on physical activity in adults and older adults with stroke. However, Iwamoto et al. [22] reported significant improvements in upper extremity dressing (*p* = 0.046) and arm function (*p* = 0.023) in participants who received physical exercise combined with OT. This intervention lasted 2 weeks with 5 sessions per week, each lasting 40 min. Long et al. [23] reported significant gains in upper limb function (Fugl-Meyer assessment, *p* < 0.001) and functional performance (*p* < 0.001) after 3 weeks of VR training (5 weekly sessions, 45 min each). Similarly, Marques-Sule et al. [27] observed significant improvements in balance and ADL performance, measured by the Timed Up and Go (*p* < 0.001), Berg Balance Scale (*p* < 0.01), and Frenchay Activity Index (*p* < 0.001), following 8 weeks of VR training with the Nintendo Wii (Nintendo, Kyoto, Japan; 3 weekly sessions, 60 min each).

### 3.9. Certainty of Evidence

The certainty of evidence was insufficient to support definitive recommendations for interventions aimed at improving ADLs, cognitive function, and physical function in middle-aged and older people with chronic stroke. Although some studies demonstrated promising results, the overall findings underscore the need for further research to draw clearer conclusions and guide evidence-based interventions in this population (Table 3).

### 3.10. Adverse Effects and Adherence

The studies included in this systematic review with meta-analysis reported adequate adherence by participants (*p* < 0.367). A 92% completed the intervention; no adverse events occurred. This suggests that these interventions are both well tolerated and feasible for stroke patients and support their potential for widespread use in similar populations.

## 4. Discussion

Results from several studies suggest that a variety of interventions can improve activities of daily living, cognitive function and physical activity in people with stroke; however, the evidence is mixed due to variability in assessment. OT, functional electrical stimulation, non-invasive brain training and VR training appear to improve ADL, while interventions targeting cognitive and motor skills also contribute to improved democracy. Although cognitive outcomes are inconsistent and there is a period of assessment, VR and a variety of OT interventions have shown significant improvements in attention, memory and general cognition. The benefits of physical activity are particularly positive, with interventions such as combined limb training, real physical therapy and dual cognitive-motor skills demonstrating significant improvements in motor skills, balance and gait. Overall, these results highlight the potential of comprehensive, multidisciplinary rehabilitation strategies to promote recovery in people with stroke and highlight the need for further research to establish the process and improve outcomes.

### 4.1. Activities of Daily Living

A systematic review showed a significant improvement in COPM in favor of EG compared with CG. Legg et al. [30] found that OT for stroke significantly improved ADL performance scores (*p* = 0.02), and participants achieved greater independence in long-term ADL activities (*p* = 0.005). Elaifi et al. [31] reported the results of an electronic study performed in the first two months after stroke and showed a significant improvement in motor function and ADL independence (*p* < 0.06). In their meta-analysis, Ahmed et al. [32] found that all methods except fixed theta burst exercise and cathodal transcranial direct current stimulation did not cause brain stimulation to be more effective than sham stimulation in improving ADL (*p* = 0.005). Gao et al. [33] reported a significant effect of repeated transcranial magnetic stimulation with ADL skills after stroke (*p* < 0.05). Leong et al. [34] also found that hybrid VR therapy could effectively improve muscle function (*p* = 0.0005) and ADL performance (*p* = 0.003). These findings suggest that OT, electrical stimulation, noninvasive brain stimulation, and VR training interventions may improve motor function and activities of daily living. Combining physical training with cognitive skills may help people with stroke recover by increasing their independence in activities of daily living.

### 4.2. Cognitive Function

The cognitive function analysis yielded mixed results, and several studies found no significant differences between EG and CG. Meta-analysis was not feasible due to variability in assessment methods. While Iwamoto et al. [22] found no significant changes in the Mini-Mental State after Hybrid Assistive Limb training combined with OT, VR training interventions reported by Long et al. [23] and Faria et al. [26] showed significant improvements in attention and memory (*p* < 0.05); this is similar to Chen et al. [35] reported significant cognitive gains (*p* < 0.05), as assessed by the Mini-Mental State, Montreal Cognitive Assessment, and Loewenstein Occupational Therapy Cognitive Assessment. In the same way, O’Donoghue et al. [36] highlighted the efficacy of multicomponent and physical activity-based interventions, reporting significant improvements in overall cognitive functioning (*p* = 0.04) and functional status (*p* = 0.05). On the other hand, Loetscher et al. [37] observed no convincing effects of cognitive rehabilitation on attention deficits (*p* = 0.41), although it significantly improved divided attention immediately after treatment (*p* < 0.001). These findings suggest that VR training and multicomponent OT hold promise for cognitive recovery, though standardization of assessment tools and intervention protocols is needed.

### 4.3. Physical Function

Although a meta-analysis was planned, differences between tests were not included in the results. However, some studies show the benefits of physical intervention. Iwamoto et al. [22] showed that the use of hybrid leg support with overextension improved body wear (*p* = 0.046) and arm use (*p* = 0.023); this is similar to Long et al. [23] who found improvements in Fugl-Meyer assessment (*p* < 0.000) and performance (*p* < 0.000) with VR training, while Marques-Sule et al. [27] found that balance and physical activity were significantly better when using the Nintendo Wii (Nintendo, Kyoto, Japan; *p* < 0.001). Cognitive motor dual-task intervention (CMDT) also yielded significant results. Zhou et al. [38] reported significant improvements in walking balance (*p* = 0.01), walking ability (*p* < 0.0001) and upper extremity function (*p* < 0.001). Similarly, He et al. [39] highlighted the effectiveness of two travel programs in improving one travel program (*p* < 0.04). These findings highlight the potential of CMDT and targeted physical interventions to improve exercise performance in stroke treatment.

### 4.4. Limitations and Strengths

This systematic review and meta-analysis have several limitations. First, differences in intervention design, including differences in dose, duration and type of activity, make it difficult to generalize the findings. Second, the absence of statistical significance in cognitive and physical activity in many studies limits the strength of the conclusion regarding positive effects. Finally, differences in cognitive assessment, particularly with instruments such as the Mini-Mental State and the Montreal Cognitive Assessment, highlight the need for consistency and standardization in previous research.

Despite these limitations, the systematic review also has notable strengths. It provides a better understanding of the potential of integrated therapy for people with stroke by including a variety of intervention methods, such as VR training, robot-assisted therapy, and cognitive-physical therapy programs. In addition, all studies were RCTs with good methodology, increasing the reliability of the study results. A detailed description of the intervention process, including dosage, session duration, and frequency, also supports the replication of successful strategies and demonstrates evidence-based practice.

### 4.5. Practical Applications

This excellent review with meta-analysis highlights the urgent need to develop intervention strategies that can improve activities of daily living and cognitive and physical functioning in stroke patients. This study demonstrates the great potential of integrating technologies such as VR training and robot-assisted therapy into traditional medical practice. These techniques demonstrated their effectiveness as tools that can be incorporated into clinical programs, demonstrating significant improvements in ADL performance, length, and frequency.

However, the inconsistency observed in cognitive outcomes suggests a necessity for standardizing intervention parameters, including intensity, duration, and frequency. By doing so, practitioners can optimize the effectiveness of these interventions. Integrating hybrid programs combining physical and cognitive components may enhance patient engagement and outcomes. Such programs facilitate comprehensive rehabilitation and promote patient motivation and adherence to therapy by making the sessions more engaging and interactive.

Long-term follow-up studies are crucial to assess the sustainability of gains achieved in ADLs and cognitive functions. These studies are also vital for refining intervention strategies to ensure they are culturally tailored and relevant, especially given the increasing prevalence of chronic stroke worldwide. By focusing on personalized rehabilitation strategies, healthcare providers can better address each patient’s unique needs, thereby maximizing the potential for recovery and improving overall quality of life.

## 5. Conclusions

The study found significant improvements in ADL performance, as measured by the COPM, while no significant improvements were observed in cognitive or physical functions among middle-aged and older adults with chronic stroke; this suggests that OT interventions incorporating cognitive, motor, and technological elements, such as VR training and non-invasive brain stimulation, hold promise for enhancing ADLs, cognitive, and physical functions. Future research should prioritize the specification of the dose, duration, and components of these interventions to better understand their impact and facilitate replication in subsequent studies and clinical applications. Virtual simulations of daily activities can empower patients by promoting independence and facilitating social reintegration. Additionally, incorporating neuroscientific methods, such as non-invasive brain stimulation, can enhance brain plasticity and functional recovery, offering a comprehensive approach to stroke rehabilitation.

These innovative approaches not only provide new directions for post-stroke rehabilitation but also emphasize the importance of evidence-based practice tailored to individual patient needs. The development of specific intervention protocols based on scientific evidence is essential to ensure that treatments are effective, adaptable, and aligned with the unique needs of each patient. By doing so, healthcare professionals can guarantee that interventions lead to improved functional outcomes and quality of life for stroke survivors. The findings of this study highlight the potential of combining traditional therapeutic methods with cutting-edge technology to create a holistic and practical rehabilitation framework.

## Figures and Tables

**Table 1:** Selection criteria used in the systematic review.

Category	Inclusion	Exclusion
Population	Studies that were conducted in the chronic stroke population with a mean age of 45 years or more.	Studies with populations whose central pathology is other than a CVA (brain injury, multiple sclerosis, ALS, or other neurological or neurodegenerative condition) and under 45 years of age.
Intervention	Studies involving interventions or programs of OT from 4 weeks onwards.	Studies whose focus of intervention was not related to OT interventions
Comparison	Interventions with active or inactive control groups.	Lack of reference and/or follow-up data. Absence of control group.
Outcome	At least one assessment of ADLs or cognitive function or physical function.	Does not present any follow-up assessment.
Study design	Randomized controlled trial studies.	Non-randomized, cross-sectional, retrospective, and prospective controlled studies.

ADL: Activities of Daily Living; ALS: Amyotrophic Lateral Sclerosis; CVA: Cerebrovascular Accident.

**Table 2:** Characteristics of the included randomized clinical trials.

Authors	Country or Multicenter	Groups (*n*)	Mean Age (Years)	Type of Intervention and Control Group	Training Volume			Training Intensity	Assessments	Main Outcomes
					Weeks	Frequency (Sessions/Week)	Session Duration (Minutes)			
[21]	South Korea	Total: 43 EG: 20 CG: 23	52.6 years old	EG: CO-OP approach CG: Conventional occupational therapy	5	NR	NR	NR	COPM and PQRS	COPM-Performance: ↑ *p* < 0.001 COPM-Satisfaction: ↑ *p* < 0.001 PQRS: ↑ *p* < 0.001
[22]	Japan	Total: 30 EG: 15 CG: 15	61.2 years old	EG: received training with the HAL in combination with occupational therapy CG: received only occupational therapy without the HAL	2	5	40 min	NR	Br-stage, FIM and MAL	Both groups: ↔ Upper Limb Motor Function (Br-stage) (*p* = 0.003) EG: ↑ Dressing of Upper Body (FIM) (*p* = 0.046) ↑ Amount of Use-Putting Arm Through Sleeve (MAL) (*p* = 0.023) ↑ Quality of Movement (*p* = 0.043) ↑ Motor Sub score (FIM) (*p* = 0.009) CG: ↔ No significant changes in ADL assessments (*p* > 0.05)
[23]	China	Total: 52 EG: 25 CG: 27	53 years old	EG: VR training using the Doctor Kinetic system CG: Standard rehabilitation without VR	3	5	45 min	NR	MBI and FMA-UE	Both groups: ↔ ADL (MBI) (*p* = 0.030) EG: ↑ MBI (*p* < 0.000) ↑ FMA-UE (*p* < 0.000) CG: ↑ MBI (*p* < 0.000) ↑ FMA-UE (*p* < 0.000)
[24]	Canada	Total: 21 EG: 10 CG: 11	57.4 years old	Determine the potential effectiveness of OPC-Stroke. EC: OPC–Stroke plus usual care. CG: Usual care only.	16	10	NR	NR	COPM, GSAB-DFI, MoCA and RNLI	EG: ↑ COPM Performance (*p* < 0.001) ↑ COPM Satisfaction (*p* < 0.001) ↔ GSAB–DFI (*p* = 0.731) ↔ MoCA (*p* = 0.070) ↔ RNLI (*p* = 0.231) CG: ↑ COPM-Performance (*p* < 0.001) ↑ COPM-Satisfaction (*p* < 0.001) ↔ GSAB–DFI (*p* = 0.819) ↔ MoCA (*p* = 0.065) ↔ RNLI (*p* = 0.212)
[25]	South Korea	Total: 48 EG: 24 CG: 24	58.3 years old	EG: SG using the RAPAEL Smart Glove™ for distal upper extremity rehabilitation CG: CON involving standard range of motion and strengthening exercises, tabletop activities, and training for ADLs	4	5	30 min	No reported	FM, JTT and SIS	EG: ↔ FM-total (T0: 53.4 ± 1.8, T2: 58.3 ± 1.7, *p* < 0.001) ↑ FM-total (T3: 58.5 ± 1.7, *p* = 0.001) ↑ FM-prox (T2: 32.5 ± 0.9, *p* = 0.001) ↑ FM-prox (T3: 32.7 ± 0.9, *p* = 0.001) ↑ JTT-total (*p* = 0.032) ↑ JTT-gross (*p* = 0.025) ↑ Composite SIS (T2: 36.7 ± 10.0, *p* = 0.001) CG: ↔ FM-total (T0: 48.2 ± 2.6, T2: 49.6 ± 2.7, *p* = 0.512) ↔ FM-total (T3: 49.5 ± 2.7, *p* = 0.592) ↔ FM-prox (T2: 28.9 ± 1.4, *p* = 0.538) ↔ JTT-total ↔ JTT-gross ↔ Composite SIS (T2: 1.9 ± 10.5, *p* = 0.856)
[26]	Portugal	Total: 18 EG: 9 CG: 9	55.1 years old	EG: VR training simulation of activities of daily living (Reh@City) CG: Time-matched conventional cognitive training (paper-and-pencil tasks).	4–6	3–4	20 min	NR	ACE, MMSE and SIS	Both groups: ↔ ADL (SIS) (*p* = does not specify) EG: ↑ Global cognitive functioning (ACE) (*p* = 0.014) ↑ Attention (*p* = 0.040) ↑ Memory (*p* = does not specify) ↑ Visuo-spatial abilities (p = does not specify) ↑ Physical domain (Strength) (*p* = 0.017) ↑ Physical domain (Mobility) (*p* = 0.012) ↑ MMSE (*p* = 0.050) CG: ↓ Verbal fluency (*p* = does not specify) ↔ Memory self-report (*p* = does not specify) ↔ Social participation (*p* = does not specify) ↔ MMSE (*p* = does not specify)
[27]	Spain	Total: 29 EG: 15 CG: 14	66.4 years old	EG: VR training WiiG CG: CPTG	8	3	60	Moderate	BBS, BI, FAI, POMA, and TUG	BBS: ↑ *p* < 0.01 BI: ↑ *p* < 0.01 FAI: ↑ *p* < 0.001 POMA-Total: ↑ *p* < 0.001 POMA-Gait: ↑ *p* < 0.01 POMA-Balance: ↑ *p* < 0.01 TUG: ↑ *p* < 0.001
[28]	South Korea	Total: 33 EG: 15 CG: 15	Middle age: 52.6 years old	EG: CO-OP approach CG: Conventional occupational therapy	8	5	45	Moderate	K-MBI and MFT	K-MBI: ↑ *p* < 0.001 MFT: ↑ *p* = 0.050
[29]	Israel	Total: 66 EG: 33 CG: 33	Mean age: 64.6 years old	EG: FaCoT intervention CG: Standard care	12	3	60	Moderate	COPM	COPM-Performance: ↑ *p* < 0.001 COPM-Satisfaction: ↑ *p* < 0.001

ACE: Addenbrooke Cognitive Examination; ADL: Activities of Daily Living; BBS: Berg Balance Scale; BI: Barthel Index; Br-stage: Brunnstrom Recovery Stage; CA: Canada; CG: Control Group; CO-OP: Cognitive Orientation to Occupational Performance; COPM: Canadian Occupational Performance Measure; CPTG: Conventional Physical Therapy Group; EG: Experimental Group; FaCoT: Functional and Cognitive Occupational Therapy; FAI: Frenchay Activity Index; FIM: Functional Independence Measure; FM: Fugl–Meyer assessment; FMA-UE: Fugl-Meyer Assessment-Upper Extremity; GSAB–DFI: Goals Systems Assessment Battery–Directive Functions Indicators; HAL: Hybrid Assistive Limb; JTT: Jebsen–Taylor Hand Function Test; K-MBI: Korean Modified Barthel Index; MAL: Motor Activity Log; MBI: Modified Barthel Index; MFT: Manual Function Test; MMSE: Mini-Mental State Examination; MoCA: Montreal Cognitive Assessment; NR: Not reported; POMA: Tinetti Performance-Oriented Mobility Assessment; PQRS: Performance Quality Rating Scale; RCT: Randomized Clinical Trial; RNLI: Reintegration to Normal Living Index; SG: Smart Glove; SIS: Stroke Impact Scale; TUG: Timed Up and Go; VR: Virtual Reality. ↑: Indicates significant improvements in the EG. ↔ Indicates that there are no significant improvements in the EG. ↓: Indicates significant deterioration in the EG.

**Table 3:** Methodological Quality Assessment using the GRADEpro tool.

Certainty of Evidence							Nº of Patients		Effect		Certainty	Importance
Nº of Studies	Study Design	Risk Assessment	Inconsistency	Indirect Evidence	Vagueness	Other Considerations	[Conventional Therapy Plus Virtual Reality]	[Conventional Therapy]	Relative (95% CI)	Absolute (95% CI)		
**Comparison of cognitive orientation to daily occupational performance and conventional occupational therapy on occupational performance in individuals with stroke: A randomized controlled trial**												
1	RCT	Very serious	It is not serious	It is not serious	It is not serious	None	20/43 (46.5%)	23/43 (53.5%)	Not estimable		++ Go down	IMPORTANT
**Combination of Exoskeletal Upper Limb Robot and Occupational Therapy Improve Activities of Daily Living Function in Acute Stroke Patients**												
1	RCT	Serious	It is not serious	It is not serious	It is not serious	None	15/30 (50.0%)	15/30 (50.0%)	Not estimable		+++ Moderate	IMPORTANT
**Effects of Virtual Reality Training on Occupational Performance and Self-Efficacy of Patients with Stroke: A Randomized Controlled Trial**												
1	RCT	Serious	It is not serious	It is not serious	It is not serious	None	25/52 (48.1%)	27/52 (51.9%)	Not estimable		+++ Moderate	IMPORTANT
**Occupational Performance Coaching for Stroke Survivors: A Pilot Randomized Controlled Trial**												
1	RCT	Serious	It is not serious	It is not serious	It is not serious	None	10/21 (47.6%)	11/21 (52.4%)	Not estimable		+++ Moderate	IMPORTANT
**Effects of virtual reality-based rehabilitation on distal upper extremity function and health-related quality of life: a single-blinded, randomized controlled trial**												
1	RCT	Serious	It is not serious	It is not serious	It is not serious	None	24/48 (50.0%)	24/48 (50.0%)	Not estimable		+++ Moderate	IMPORTANT
**Benefits of Virtual Reality-Based Cognitive Rehabilitation Through Simulated Activities of Daily Living: A Randomized Controlled Trial with Stroke Patients**												
1	RCT	Very serious	It is not serious	It is not serious	It is not serious	None	9/18 (50.0%)	9/18 (50.0%)	Not estimable		++ Go down	IMPORTANT
**Effectiveness of Nintendo Wii and Physical Therapy in Functionality, Balance, and Daily Activities in Chronic Stroke Patients**												
1	RCT	It is not serious	It is not serious	It is not serious	It is not serious	None	15/29 (51.7%)	14/29 (48.3%)	Not estimable		++++ High	IMPORTANT
**The Effect of Task-Oriented Activities Training on Upper-Limb Function, Daily Activities, and Quality of Life in Chronic Stroke Patients: A Randomized Controlled Trial**												
1	RCT	Serious	It is not serious	It is not serious	It is not serious	None	15/30 (50.0%)	15/30 (50.0%)	Not estimable		+++ Moderate	IMPORTANT
**Effectiveness of the Functional and Cognitive Occupational Therapy (FaCoT) Intervention for Improving Daily Functioning and Participation of Individuals with Mild Stroke: A Randomized Controlled Trial**												
1	RCT	Very serious	It is not serious	It is not serious	It is not serious	None	33/66 (50.0%)	33/66 (50.0%)	Not estimable		++ Low	IMPORTANT

## Data Availability

Data are contained within the article.
